# A Functional Bacterium-to-Plant DNA Transfer Machinery of *Rhizobium etli*

**DOI:** 10.1371/journal.ppat.1005502

**Published:** 2016-03-11

**Authors:** Benoît Lacroix, Vitaly Citovsky

**Affiliations:** Department of Biochemistry and Cell Biology, Stony Brook University, Stony Brook, New York, United States of America; Michigan State University, UNITED STATES

## Abstract

Different strains and species of the soil phytopathogen *Agrobacterium* possess the ability to transfer and integrate a segment of DNA (T-DNA) into the genome of their eukaryotic hosts, which is mainly mediated by a set of virulence (*vir*) genes located on the bacterial Ti-plasmid that also contains the T-DNA. To date, *Agrobacterium* is considered to be unique in its capacity to mediate genetic transformation of eukaryotes. However, close homologs of the *vir* genes are encoded by the p42a plasmid of *Rhizobium etli*; this microorganism is related to *Agrobacterium*, but known only as a symbiotic bacterium that forms nitrogen-fixing nodules in several species of beans. Here, we show that *R*. *etli* can mediate functional DNA transfer and stable genetic transformation of plant cells, when provided with a plasmid containing a T-DNA segment. Thus, *R*. *etli* represents another bacterial species, besides *Agrobacterium*, that encodes a protein machinery for DNA transfer to eukaryotic cells and their subsequent genetic modification.

## Author Summary

Since the discovery of gene transfer from *Agrobacterium* to host plants in the late 1970s, this bacterial pathogen has been widely used in research and biotechnology to generate transgenic plants. *Agrobacterium*’s infection process relies on a set of virulence proteins that mediate the transfer of a segment of its own DNA (T-DNA) into the host cell genome. To date, *Agrobacterium* is believed to be the only prokaryote with the capability of cross-kingdoms gene transfer. However, homologs of the *Agrobacterium*’s virulence proteins are found in some symbiotic plant-associated bacterial species, belonging to the *Rhizobium* genus. Here we show that one of these species, *Rhizobium etli*, encodes a complete set of virulence proteins and is able to mediate transfer and integration of DNA into host-plant cell genome, when provided with a T-DNA. This is the first time that a bacterium-to-plant DNA transfer machinery encoded by a non-*Agrobacterium* species is shown to be functional.

## Introduction

The Rhizobiales order contains many species of plant-associated bacteria, such as the related genera *Agrobacterium* and *Rhizobium*. Phylogenetic analyses based on 16S rDNA sequences led to the idea that *Agrobacterium* and *Rhizobium* could be regrouped into one genus [1]. Yet their lifestyles are very different. *Agrobacterium* comprises species that are often, but not always [2, 3], pathogenic and can genetically transform their host plant cells by transferring a segment of their own plasmid, the T-DNA, and induce neoplastic growths that synthesize small molecules used as nutrients by the bacteria [4–6]. This *Agrobacterium* capability to modify genetically their host cells is widely used in research and biotechnology for generating transgenic plants [7] as well as fungi [8]. In contrast, *Rhizobium* belongs to a group of very diverse symbiotic bacteria (collectively termed rhizobia) that form nitrogen-fixing nodules on the roots of legume plants [9–12]. *Rhizobium* and *Agrobacterium* species have complex genomes composed of one or two chromosomes and several plasmids [13–16]; the chromosomes are designed as “core” components defining the species as opposed to the “accessory” components that are the plasmids [17]. The outcome of interactions of these bacteria with plants is essentially determined by large specialized plasmids, the tumor inducing (Ti) plasmid for *Agrobacterium*, and symbiotic (pSym) plasmid for *Rhizobium*. Indeed, introducing an *Agrobacterium* Ti plasmid into some rhizobia species resulted in virulent bacteria capable of inducing tumors in host plants [18]. In general, rhizobia species are known to gain T-DNA transfer ability only when provided with the virulence (*vir*) genes [4, 5] of the *Agrobacterium* Ti plasmid [19, 20]. *Rhizobium*, therefore, is thought to possess chromosomal, but not plasmid-based factors required for plant genetic transformation, and because of that lack endogenous DNA transfer capacity.

Intriguingly, however, many *Rhizobium* species harbor different sets of homologs of the *Agrobacterium vir* genes; specifically, *R*. *etli* carries a complete set of *vir* genes [15, 21] whereas the closely related *R*. *leguminosarum* lacks such degree of homology. Here, we show that *R*. *etli* can independently mediate functional DNA transfer and stable genetic transformation of plant cells, when provided with a plasmid containing a T-DNA segment. Thus, *R*. *etli* represents another bacterial species, in addition to *Agrobacterium*, capable of genetic modification of plants.

## Results

### *vir* gene homologs in *R*. *etli*

**Fig 1:**
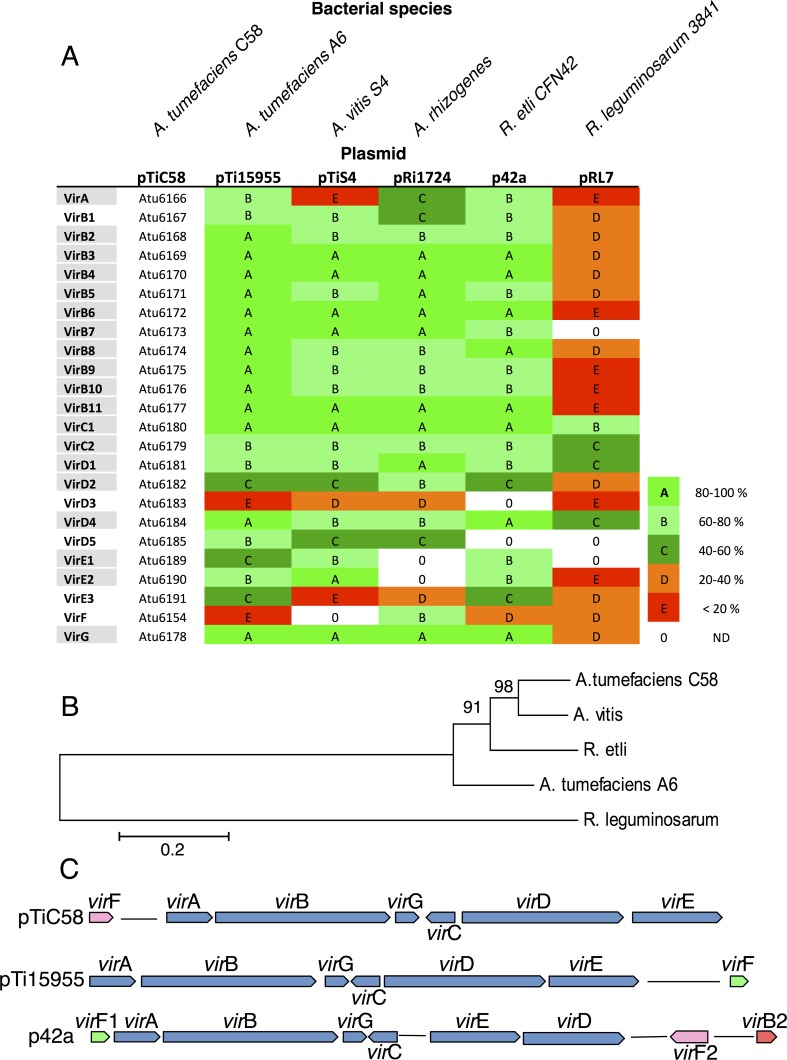
Sequence comparison of *Agrobacterium* and *Rhizobium* Vir proteins and organization of the *vir* gene regions. A, percentage of sequence identity between orthologs of Vir proteins in *A*. *tumefaciens* A6, *A*. *vitis* S4, *A*. *rhizogenes*, *R*. *etli* CFN42, and *R*. *leguminosarum* bv. viciae 3841, with *A*. *tumefaciens* C58 as reference; color codes correspond to percentage of identity as indicated, grey background under the protein name indicates the proteins essential for T-DNA transfer by *Agrobacterium*. B, phylogenetic tree of VirE2 protein orthologs from the bacterial species listed in A, except for *A*. *rhizogenes* that does not encode VirE2. The evolutionary history was inferred using the Neighbor-Joining method [44]. The optimal tree with the sum of branch length = 2.23186255 is shown. The percentage of replicate trees, in which the associated taxa clustered together in the bootstrap test (1,000 replicates) is shown next to the branches [45]. The tree is drawn to scale, with branch lengths in the same units as those of the evolutionary distances used to infer the phylogenetic tree. The evolutionary distances were computed using the Poisson correction method [46] and are in the units of the number of amino acid substitutions per site. The analysis involved 5 amino acid sequences. All positions containing gaps and missing data were eliminated. There were a total of 509 positions in the final dataset. Evolutionary analyses were conducted in MEGA6 [29]. Bar = 0.2 amino acid substitution per site. C, organization of *vir* gene regions of *A*. *tumefaciens* C58, *A*. *tumefaciens* A6 and *R*. *etli* CFN42.

Sequencing of the *R*. *etli* CFN42 genome revealed that it encodes a complete set of virulence (Vir) proteins encoded by the *vir* genes [15, 22]. Indeed, Fig 1A shows that all the essential Vir proteins encoded by the p42a plasmid of *R*. *etli* exhibit a high level of homology with their counterparts from different *Agrobacterium* Ti plasmids, except for the VirD3 and VirD5 proteins, which are non-essential for DNA transfer. Phylogenetic analysis demonstrated that the Vir proteins of *R*. *etli* and *Agrobacterium* are very close to each other, as exemplified for VirE2 (Fig 1B). In contrast, the putative Vir protein orthologs of *R*. *leguminosarum* only share a relatively weak homology, i.e., usually less than 40% identity, with *Agrobacterium*. Fig 1C shows that, within the p42a plasmid of *R*. *etli*, the *vir* genes are grouped in a cluster, forming a virulence region that is similar in many ways with the *vir* region of *Agrobacterium* Ti-plasmids, but it also displays some notable differences. Specifically, the organization of the “core” of the *vir* region—the *vir*A, *vir*B, *vir*G, *vir*C, *vir*D, and *vir*E operons—is nearly identical, but the order of the *vir*D and *vir*E operons is inverted in *R*. *etli*. In addition, in *R*. *etli*, the *vir*B2 coding sequence is not part of the *vir*B operon, but is located at a distant locus on the same plasmid, and two *vir*F homologs are present, *vir*F1 and *vir*F2, which are related to the *vir*F genes from octopine (A6) and nopaline (C58) *Agrobacterium* strains, respectively. The presence of many transposase insertion sequences in the vicinity of the *vir* cluster of *R*. *etli* [15] may explain the rearrangements in the organization of the *vir* region. In *R*. *leguminosarum*, the organization of the *vir* region located on the pRL7 plasmid appears to be scrambled, with several operons having been duplicated (see the pRL7 map in the KEGG database, http://www.genome.jp/kegg/). Although other *Rhizobium* species, such as *R*. *mesoamericanum* and *R*. *tropici*, contain homologs of several *vir* genes (S1 Table), a high level of homology with all essential *vir* genes is found only in *R*. *etli*. Whereas a complete *vir* region is present in the *R*. *etli* p42a plasmid which is homologous to the *Agrobacterium vir* genes, we could not detect homologies to any of the *Agrobacterium* T-DNA sequences; specifically, our search for T-DNA-specific oncogenes and opine synthesis genes and for the T-DNA border sequences did not yield significant homology.

### Transient and stable genetic transformation of plants by *R*. *etli*

**Fig 2:**
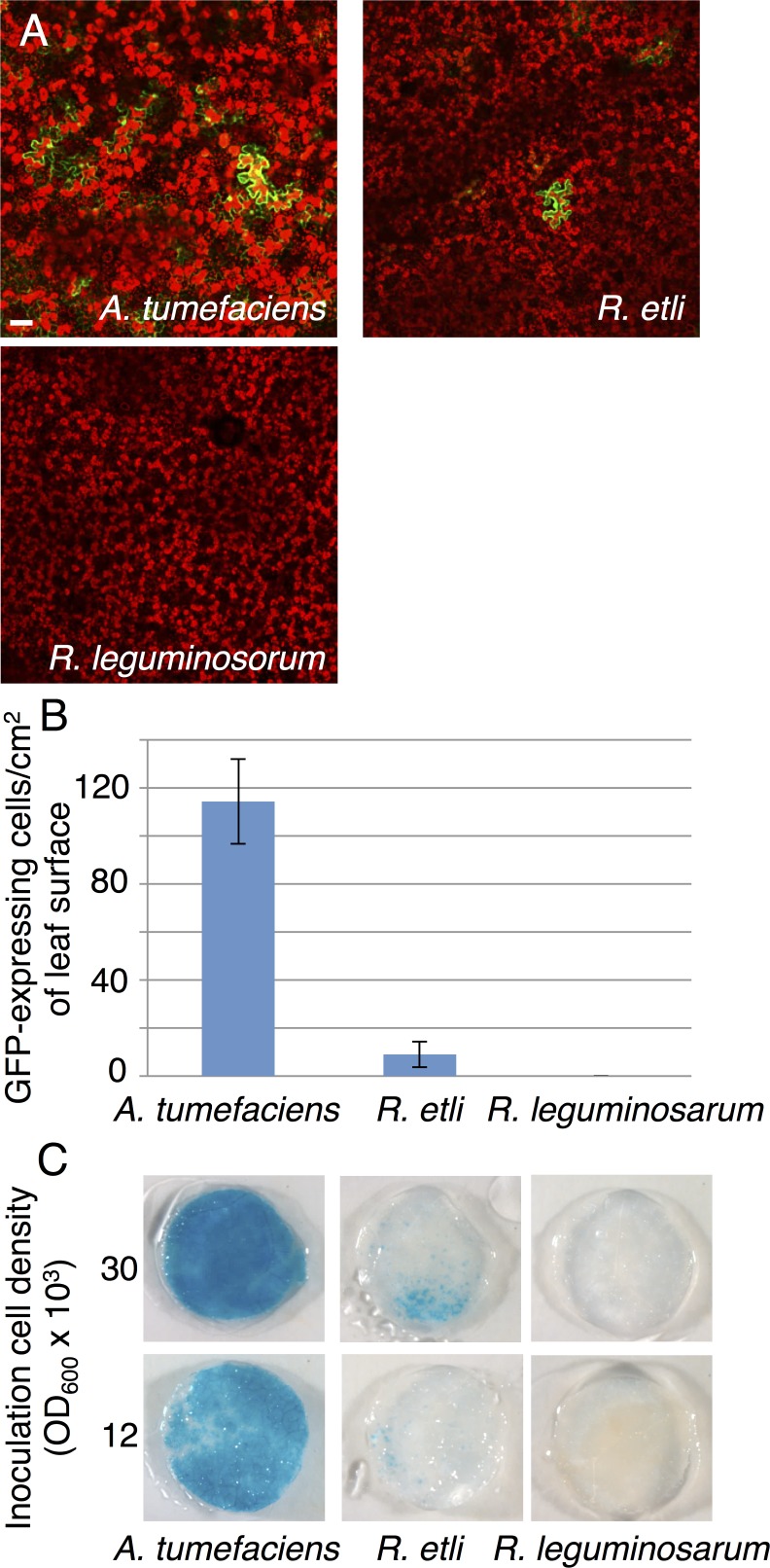
Transient expression of reporter genes delivered to plants by *R*. *etli*. A, expression of GFP in *N*. *benthamiana* leaf tissue 3 days after infiltration with *A*. *tumefaciens* EHA105, *R*. *etli* CE3 and *R*. *leguminosarum* 3841, harboring the pCB302T-GFP plasmid. Plastid autofluorescence is in red, GFP fluorescence is in green. Bar = 50 μm. B, numbers of GFP-expressing cells per cm^2^ of infiltrated leaf surface. C, GUS activity in leaf discs from infiltrated *N*. *benthamiana* leaves 3 days after infiltration with the indicated dilutions of cultures of *A*. *tumefaciens* EHA105, *R*. *etli* CE3 or *R*. *leguminosarum* 3841, harboring the pBISN1 plasmid.

To examine potential functionality of the *vir* genes of *R*. *etli*, we introduced into *R*. *etli* cells a plasmid that harbors a T-DNA sequence with reporter genes *gfp* or *gus*-int, and selection gene *npt*II but lacks any *vir* sequences. This strain was then tested for its ability to promote transient T-DNA expression in plant cells and generate stably transformed transgenic plants, and compared to *A*. *tumefaciens* EHA105, one of the standard strains for plant genetic transformation [24]. After infiltration of *Nicotiana benthamiana* leaves with *R*. *etli*, expression of both GFP (Fig 2A and 2B) and β–glucuronidase (GUS) reporters (Fig 2C) was consistently observed in the inoculated plant tissues, although expression levels with *R*. *etli* were about ten times lower than those with *A*. *tumefaciens* (Fig 2B). Thus, *R*. *etli* was able to transfer to plant cells DNA that subsequently could be expressed. In contrast, in similar experiments performed with *R*. *leguminosarum*, transient expression of the reporter *gfp* or *gus*-int genes was never observed (Fig 2).

**Fig 3:**
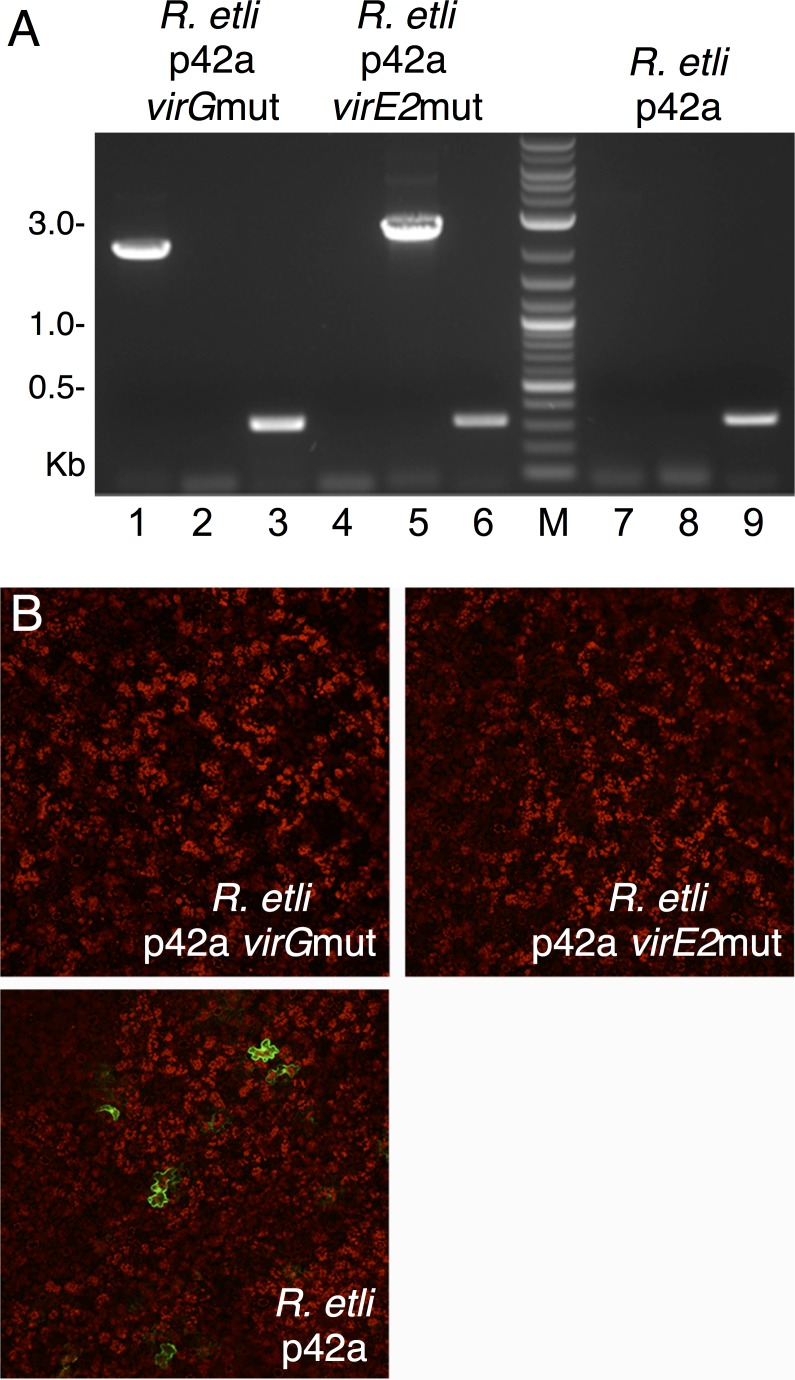
DNA delivery to plants by *R*. *etli* depends on *vir* genes of p42a. A, PCR analysis of the presence of mutagenic *gusA* in the *vir*G and *vir*E2 genes of the mutant p42a plasmids. Lanes 1–3, *R*. *etli* carrying p42a with mutant *vir*G (*vir*Gmut). Lanes 4–6, *R*. *etli* carrying p42a with mutant *vir*E2 (*vir*E2mut). Lanes 7–9, *R*. *etli* carrying wild-type p42a. DNA samples were amplified with primers specific for *gusA* and *vir*G (lanes 1, 4, 7), *gusA* and *vir*E2 (lane 2, 5, 8), or chromosomal DNA (lanes 3, 6, 9). Lane M, molecular size markers. Numbers on left indicate molecular sizes in thousands of base pairs (Kb). B, Transient expression of GFP in *N*. *benthamiana* leaf tissue 3 days after infiltration with *R*. *etli* carrying p42a *vir*Gmut, p42a *vir*E2mut, or wild-type p42a. Plastid autofluorescence is in red, GFP fluorescence is in green. Bar = 50 μm.

That *R*. *leguminosarum*—which is very closely related to *R*. *etli*, except for the *vir* region—is unable to effect genetic transformation suggests that it is the *vir* genes that are required for the T-DNA transfer by *R*. *etli*. We tested this notion directly using *R*. *etli* carrying p42a with *vir*G or *vir*E2 genes mutated by insertion of a promoterless *gusA* gene [21]. PCR-based analysis using primers specific for *gusA* and *vir*G and *vir*E2 showed that *R*. *etli* cells with the mutated p42a plasmids, i.e., p42a *vir*Gmut and p42a *vir*E2mut, indeed, contained the mutagenic sequences inserted in the sense orientation within the *vir*G and *vir*E2 genes. Specifically, Fig 3A shows that the reverse primer, corresponding to the 3’-end of *gusA*, and forward primers, corresponding to the 5’-ends of *vir*G and *vir*E2, amplified fragments of ca. 2.3 Kb (lane 1) and 2.8 Kb (lane 5) for the *vir*Gmut and *vir*E2mut mutants, respectively, but not for the wild-type genes in the same strains, i.e., for *vir*E2 in the *vir*Gmut strain (lane 2) and for *vir*G in the *vir*E2mut strain (lane 4). As expected, no *gusA* sequences were detected in the wild-type p42a plasmid (Fig 3A, lanes 7, 8) whereas all samples contained bacterial chromosomal DNA (Fig 3A, lanes 3, 6. 9). Neither of these plasmids was able to promote transfer and transient expression of the *gfp* reporter gene (Fig 3B). In control experiments with *R*. *etli* carrying the wild-type p42a, the *gfp* reporter was transferred to plant cells, resulting in expression of its protein product (Fig 3B, see also Fig 2A). These observations suggest that the T-DNA transfer mediated by *R*. *etli* relies on and requires its *vir* genes.

**Fig 4:**
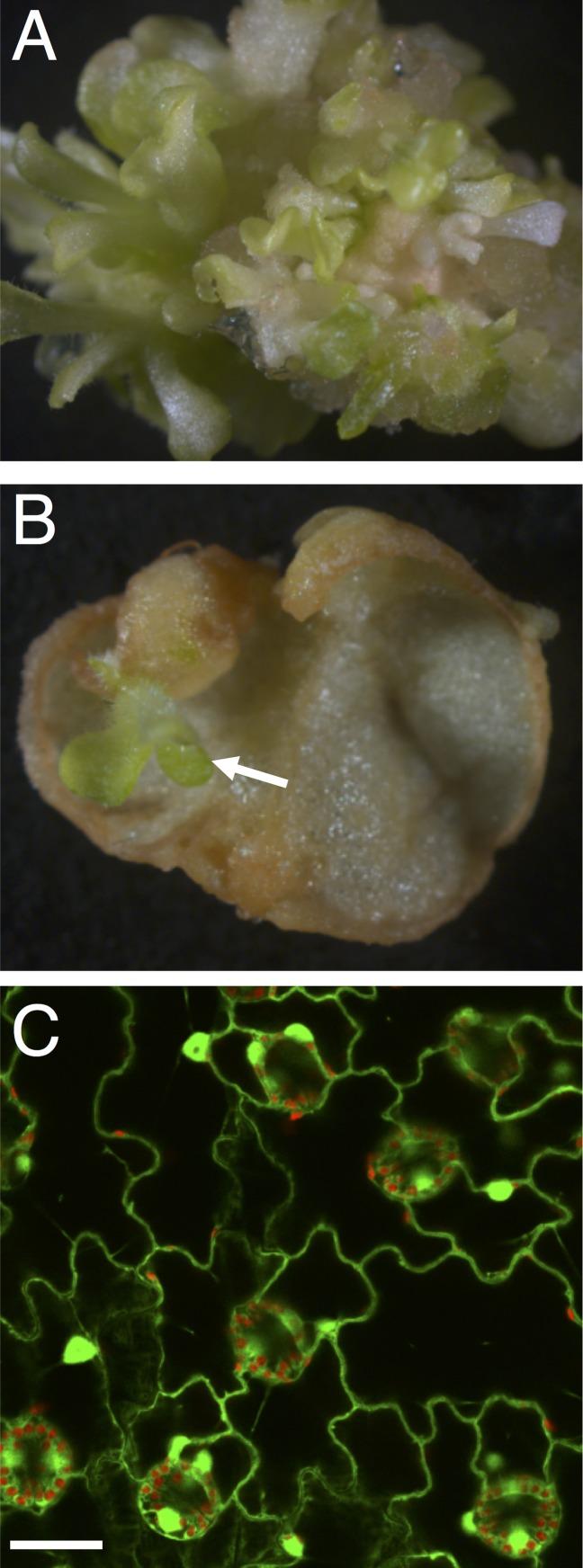
Generation of transgenic plants using *R*. *etli*. A, B, regeneration of transgenic tobacco plantlets on kanamycin selection medium, 4 weeks after inoculation with *A*. *tumefaciens* EHA105 (A) or *R*. *etli* CE3 (B), harboring pBin19-EGFP. Arrow indicates a regenerated plantlet. C, expression of GFP in leaf of transgenic plantlets generated using *R*. *etli* CE3, harboring pBin19-EGFP. Image is a projection of several confocal sections. Plastid autofluorescence is in red, GFP fluorescence is in green. Bar = 10 μm.

For stable genetic transformation, tobacco (*N*. *tabacum*) leaf discs were inoculated with *R*. *etli* or *A*. *tumefaciens* harboring a plasmid with the selection gene *npt*II encoding resistance to kanamycin as well as the *gfp* marker gene in its T-DNA. Regenerating plantlets were observed after four weeks incubation under kanamycin selection, which indicates stable genetic transformation (Fig 4A and 4B). Consistent with the transient T-DNA expression data, the genetic transformation efficiency mediated by *R*. *etli* was much lower than with *A*. *tumefaciens* (compare Fig 4A to Fig 4B). Confirming stable transgene expression in the regenerated plants, GFP was observed in a typical nucleocytoplasmic pattern in virtually all cells in leaves of one-month-old transgenic plants generated using *R*. *etli* (Fig 4C).

**Fig 5:**
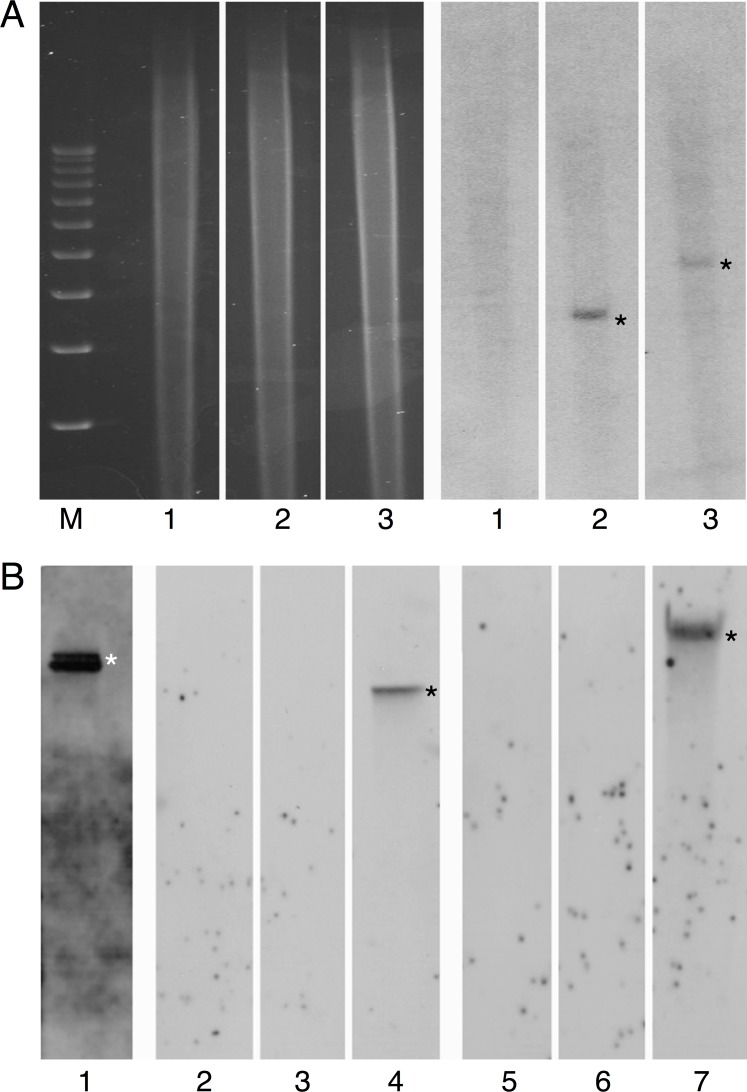
Southern blot analysis of two independent kanamycin-resistant and GFP-expressing transgenic tobacco lines. (A) Detection of the transgene. Total DNA from transgenic lines TL1 and TL2 was digested with EcoR1 and probed with the *nos* promoter region of the T-DNA segment of pBin19-RCS1-GFP. Left four lanes, Ethidium-bromide stained agarose gel prior to transfer. Right three lanes, Southern blot of the lanes shown on left. Lane M, molecular size markers (from top to bottom: 12, 11, 10, 9, 8, 7, 6, 5, 4, 3 kb); lane 1, wild-type, untransformed plant; lane 2, plant TL1; lane 3, plant TL2. Asterisks indicate the hybridized bands of the integrated T-DNA. (B) Control hybridizations. Lane 1, free pBin19-RCS1-GFP plasmid. Lanes 2, 5, wild-type, untransformed plantlet. Lanes 3, 6, transgenic line TL1. Lane 4, undigested p42a plasmid of *R*. *etli*. Lane 7, undigested chromosomal DNA of *R*. *etli*. DNA samples were digested with EcoRI (lanes 1–3, 5, 6). The blots were probed with the *nos* promoter region of the T-DNA segment of pBin19-RCS1-GFP (lane 1), the *vir*B5 gene from p42a plasmid of *R*. *etli* (lanes 2–4) or a 320-bp segment of *R*. *etli* chromosomal DNA (lanes 5–7). Asterisks indicate the hybridized bands of free pBin19-RCS1-GFP (lane 1), free undigested p42a (lane 4), or total *R*. *etli* chromosomal DNA (lane 7).

Finally, we confirmed the actual presence of the T-DNA within the genome of these transformed plants. Genomic DNA was isolated from two independent stable transgenic lines, designated TL1 and TL2, and from a wild-type, untransformed plant and analyzed by Southern blot hybridization. Specifically, the DNA samples were digested with EcoRI and hybridized them with a probe corresponding to the T-DNA right border-proximal *nos* promoter region of the T-DNA of pBin19-RCS1-GFP that has no recognition sites for EcoRI. Fig 5A shows that no hybridization signal was detected in the DNA from the wild-type plant (lane 1) whereas T-DNA-specific signal was present in the DNA of both transgenic TL1 and TL2 lines (lanes 2, 3, asterisks), suggesting a single integration site of the T-DNA within the genome of each of the tested plants. When we similarly digested purified pBin19-RCS1-GFP DNA, which has only one EcoRI recognition site in its entire sequence, a 11.9-kb band, corresponding to the linearized plasmid, was observed (Fig 5B, lane 1). Additional negative controls, which probed EcoRI-digested wild-type and transgenic plant DNA with sequences specific for the p42a plasmid (Fig 5B, lanes 2, 3) or for the *R*. *etli* chromosome (Fig 5B, lanes 5, 6) did not yield any signal. As expected, positive controls detected specific signals using undigested p42a DNA hybridized to the p42a-specific probe (Fig 5B, lane 4) and undigested *R*. *etli* chromosomal DNA hybridized with the chromosome-specific probe (Fig 5B, lane 7).

Taken together, the stable expression of two marker genes, *gfp* and *nptII*, and the physical presence of the transforming DNA in the plant genomic DNA, indicate that the T-DNA was indeed integrated into the plant genome.

## Discussion

Our results demonstrate that *R*. *etli* within its p42a plasmid contains a complete and functional *vir* region, encoding a set of Vir proteins able to mediate functional T-DNA transfer into plant cells. Whereas it has been known that the *vir* genes from *Agrobacterium* can function in several rhizobia species [18, 19], this is the first time that an endogenous virulence system encoded by a non-*Agrobacterium* species is shown to be functional in DNA transfer and stable genetic transformation.

The *vir*E2 and *vir*G mutants, which render *R*. *etli* unable to promote genetic transformation, previously have been shown to have no effect on formation of nitrogen-fixing nodules or on nodulation competitiveness [21]. Thus, the *vir* genes likely fulfill a function unrelated to symbiosis. Two factors might account for the presence of a functional *vir* region in *R*. *etli*. First, the ability to transform host plant cells may have been widespread among bacterial species in the past, and not restricted to the *Agrobacterium* genus. That we could not identify T-DNA-like sequences in *R*. *etli* suggests that *Rhizobium*-mediated plant transformation does not occur at present, although it cannot be ruled out that other *Rhizobium* strains, not yet sequenced, harbor a T-DNA. Furthermore, proteins from other rhizobia, such as *Mesorhizobium loti* R7A (see S1 Table for Vir protein sequence homologies with *M*. *loti* R7A), can be recognized by the *Agrobacterium* VirB/D4 type IV secretion system (T4SS) and exported to plant cells [25], suggesting that T4SS could substitute for the type III secretion system (T3SS) during effector protein translocation in some rhizobia species. Thus, the VirB/D4 T4SS encoded by p42a could also function to translocate protein effectors in *R*. *etli*. Second, because the p42a plasmid is transmissible between *Rhizobium* and *Agrobacterium* [21], this plasmid may belong to an “interspecies plasmid pool”, and *R*. *etli* may function as a “vector” for p42a which is then transferred to *Agrobacterium* and only then used for plant genetic transformation. It would be interesting to examine whether quorum sensing signals that activate conjugative transfer of plasmids between *Agrobacterium* cells also induce conjugation between *Rhizobium* and *Agrobacterium*. Indeed, in natural *Agrobacterium* populations, Ti-plasmids are not present in all cells [2, 3], but, in response to bacterial and plant signals via a quorum sensing mechanism, conjugative plasmid transfer can be activated [26].

The need to identify or even generate non-*Agrobacterium* bacterial species that could be used as a vector for plant genetic transformation has been emphatically articulated [27]. First, a non-*Agrobacterium* vector might be more efficient in some hosts that are difficult to transform by *Agrobacterium*. Indeed, although the efficiency of *R*. *etli* mediated transformation of *Nicotiana* species was very low compared to *Agrobacterium*, *R*. *etli* might be more efficient with other plant species, such as its native hosts. Second, several aspects of plant genetic transformation methods are legally limited by existing patents, and using a different bacterial species may help to circumvent these limitation and avoid litigation [28].

In conclusion, we demonstrate that *R*. *etli*, a symbiotic *Rhizobium* species different from the phytopathogenic *Agrobacterium*, contains the complete molecular machinery able to transfer DNA to the plant genome, which has implications for evolution and origin of the *Agrobacterium* virulence system as well as for potential utilization in biotechnology.

## Materials and Methods

### Protein sequence analysis

Protein sequences were compared using the blastp program (PubMed); the percentages of identity of full sequences were calculated as the percentage of identity corrected by the query cover percentage. VirE2 phylogenetic tree was generated using MEGA version 6 [29], via the minimum evolution method. The KEGG database release 71.0 (http://www.genome.jp/kegg/) was used to design schematic maps for the different *vir* regions.

### Bacterial strains

*R*. *etli* CE3, a streptomycin-resistant isolate of the CFN42 strain, and *R*. *leguminosarum* bv. *viciae* strain 3841 (kindly provided by Dr. Russell Carlson, University of Georgia, Athens) were grown in TY medium (5 g.L^-1^ tryptone, 3 g.L^-1^ yeast extract, and 10 mM CaCl2). *A*. *tumefaciens* strain EHA105, derived from nopaline wild-type strain C58.C1, was grown as described [30]. T-DNA containing plasmids were introduced into these *Agrobacterium* and *Rhizobium* strains using the classical CaCl_2_ protocol, with minor modifications in the case of *Rhizobium* [31]. *R*. *etli* strains, carrying p42a with mutated *vir*G and *vir*E2 genes were described previously [21].

### Plasmids

For transient expression of GFP, pCB302T-GFP was obtained by inserting the *gfp* expression cassette from pSAT1-EGFP-C1 [32] into the AgeI-BglII sites of pCB302T-MCS [33], derived from pCB302 [34]. For transient expression of GUS, pBISN1 [35], carrying an expression cassette for a *gus* reporter gene with a plant intron sequence (*gus*-int), was used. For stable transformation, the multiple cloning site of pPZP-RCS1 [36] was first introduced into the EcoRI-HindIII sites of pBin19 [37], forming pBin19-RCS1. Then, the *gfp* expression cassette from pSAT1-EGFP-C1 was inserted into the AscI site of pBin19-RCS1, resulting in pBin19-RCS1-GFP carrying both *npt*II and *gfp* expression cassettes in its T-DNA segment.

### Transient and stable plant transformation

*Agrobacterium* and *Rhizobium* strains carrying pCB302T-GFP or pBISN1 were grown 24–48 h at 28°C, and infiltrated into intact *N*. *benthamiana* leaves as described [38]. The bacterial suspension was first adjusted to OD_600nm_ 0.6 and then diluted 20 or 50 times before infiltration. Reporter gene expression was monitored three days after infiltration. For detection of GUS expression, leaf discs were excised from the infiltrated zone and subjected to the histochemical assay as described [39]. GFP expression was observed under a Zeiss LSM 5 Pascal confocal microscope at low magnification with a 10x objective; the number of GFP-expressing cells per cm^2^ of infiltrated leaf surface was counted as described [38].

Stable genetic transformation was performed using *N*. *tabacum* cv. Turk and *Agrobacterium* and *Rhizobium* strains carrying pBin19-RCS1-GFP in the classical leaf disc protocol [40]. Transgenic plantlets were selected on MS regeneration medium (30 g.L^-1^ sucrose, 8 g.L^-1^ agar, 10 mg.L^-1^ BAP, 1 mg.L^-1^ NAA) supplemented with 50 mg.L^-1^ timentin and 50 mg.L^-1^ kanamycin. Images of regenerated transgenic plantlets were recorded after 4 weeks of incubation on the regeneration/selection medium, using a Leica MZ FLIII stereoscope. Regenerated plantlets were then placed on rooting medium (30 g.L^-1^ sucrose, 8 g.L^-1^ agar) supplemented with 25 mg.L^-1^ kanamycin for one month before GFP expression in the leaves was analyzed by confocal microscopy as described above, but with a 40x objective.

### PCR analysis of mutated p42a plasmids

Total DNA was extracted from cultures of *R*. *etli* harboring p42a, p42a *vir*Gmut, or p42a *vir*E2mut [21] and PCR-amplified for 32 cycles using the primer pairs 5’ATGAAAGGTGAACGGTTGAAACAC3’/5’CCGGAATTCTCATTGTTTGCCTCCCTGCTGC3’ specific for *vir*G (RHE_PA00053) and *gusA*, 5’ATGGATCCGAAAAGCGAAGACAAT3’/5’CCGGAATTCTCATTGTTTGCCTCCCTGCTGC3’ specific for *vir*E2 (RHE_PA00061) and *gusA*, or 5’CTCCTGCGTGTCCTGATTGGC3’/5’AGCGGCGCGACGAACGTGAC3’ specific for a 320-bp segment of the *R*. *etli* chromosome between positions 109,451 and 109,770. Before proceeding with this analysis, we determined the orientation of the mutagenic *gus*A insertion in the *vir*G and *vir*E2 genes. We showed that, with forward primers corresponding to the 5’-ends of *vir*G and *vir*E2, a PCR product was observed only with the reverse primer corresponding to the 3’-end of *gusA*, but not with the primer corresponding to its 5’-end (S1 Fig), which reflects the sense orientation of *gusA* both within *vir*G and *vir*E2.

### Southern blot analysis

Total genomic DNA of wild type and transgenic tobacco plants was purified using the DNeasy plant DNA extraction kit (Qiagen) according to the manufacturer’s instructions. The purified DNA (10 μg) was digested with EcoR1 (New England Biolab) overnight. The digested DNA was resolved on a 1.0% agarose gel for 6 hours at 60 V, and DNA was transferred onto a nylon charged membrane with alkali transfer buffer [41]. For the T-DNA-specific probe, we used a 300-bp segment of the *nopaline synthase* (*nos)* promoter of the T-DNA region of pBin19-RCS1-GFP amplified using the primer pair 5’CAATATATCCTGTCAAACACTGATAG3’/5’GAAATATTTGCTAGCTGATAGTGAC3’; this probe fragment did not contain recognition sites for EcoRI. For the p42a-specific probe, we used a 240-bp segment of the *vir*B5 gene amplified using the primer pair 5’5’ATGCATGAGCTCATGAAGATGTCGAGACTAGTTAC3’/5’AAAGGATCCCCTCGTGGCGGGATACTGG3’. For the *R*. *etli* genomic probe, we used a 320-bp segment of the chromosome between positions 109,451 and 109,770 amplified using the primer pair 5’CTCCTGCGTGTCCTGATTGGC3’/5’AGCGGCGCGACGAACGTGAC3’. For Southern blot analysis of transgenic plants (Fig 5A), agarose gel electrophoresis, blotting, and detection were performed at Lofstrand Labs Ltd. (Gaithersburg, MD) using a ^32^P labeled the T-DNA-specific probe (3.26 x 10^6^ dpm/ml of hybridization buffer in a total volume of 50 ml). The hybridization was carried out for 3 days at 68°C; after washes, the membrane was autoradiographed for 17 hours with an intensifier screen at -80°C. For control experiments (Fig 5B), biotinylated probes were prepared using the biotin decalabel DNA labeling kit (Thermo Scientific); hybridization and detection were performed using the Phototope Star kit (NEB) according to the manufacturer’s instructions. Based on the 4.5 Gb size of the complex allotetraploid genome of *N*. *tabacum* [42], ca. 4 kb size of the T-DNA region of pBin19-RCS1-GFP, the DNA size-to-mass conversion ratio of 978 Mb = 1 pg (http://ebook2.worldlibrary.net/articles/C-value), and at least one T-DNA insertion per genome, we estimated that 10 μg of total transgenic plant DNA would contain ca. 9 pg of T-DNA, which is well within the detection range of the classical Southern blot analysis [43]. For comparable controls, we utilized 100 pg of purified pBin19-RCS1-GFP, 50 pg of p42a DNA, and 1 ng of *R*. *etli* chromosomal DNA.

## Supporting Information

S1 Fig PCR analysis of the orientation of the mutagenic *gusA* insertion in the *vir*G and *vir*E2 genes of the mutant p42a plasmids. Lanes 1, 2, *R*. *etli* carrying p42a with mutant *vir*G (*vir*Gmut). Lanes 3, 4, *R*. *etli* carrying p42a with mutant *vir*E2 (*vir*E2mut). DNA samples were amplified with forward primers specific for the 5’-end of *vir*G (lanes 1, 2) or *vir*E2 (lanes 3, 4) and reverse primers specific either for the 5’-end of *gusA* (lanes 1, 3) or the 3’-end of *gusA* (lane 2, 4). Lane M, molecular size markers.
(PPTX)

S1 Table Homologs of *Agrobacterium* virulence proteins in *Rhizobium mesoamericanum*, *Rhizobium tropici*, and *Mesorhizobium loti* R7A. Gray shading indicates proteins essential for *Agrobacterium* tumorigenicity [23]. OLN, ordered locus name represents the naming system for sequential assignment of an identifier to each predicted gene of a completely sequenced genome (http://www.uniprot.org/help/gene_name). Percentage of identity (Identity %) and percentage of query cover (Query Cover %) are indicated. Protein sequences were compared using the blastp program (PubMed) with the corresponding protein sequences of *Agrobacterium tumefaciens* strain C58 as reference. NS = no proteins with significant homology were identified.
(DOCX)
