# Supplementary figures and images for: A Functional Bacterium-to-Plant DNA Transfer Machinery of Rhizobium etli
Source: PLoS Pathog. 2016 Mar 11;12(3):e1005502. doi: 10.1371/journal.ppat.1005502 (PMC4788154; doi:10.1371/journal.ppat.1005502)

## Slide 1
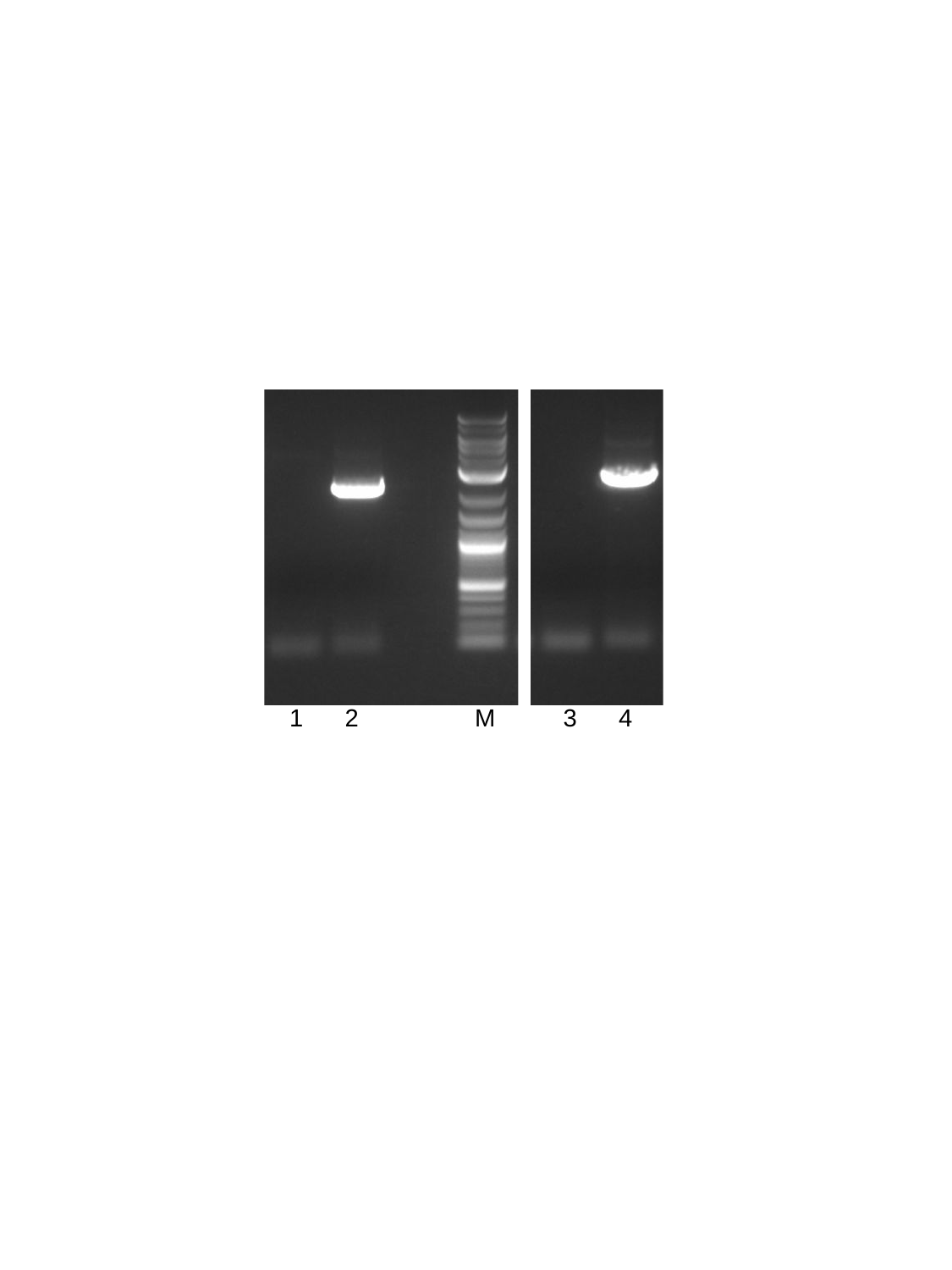

1 2
M
3 4

Supplement: S1 Fig — Lanes 1, 2, R. etli carrying p42a with mutant virG (virGmut). Lanes 3, 4, R. etli carrying p42a with mutant virE2 (virE2mut). DNA samples were amplified with forward primers specific for the 5’-end of virG (lanes 1, 2) or virE2 (lanes 3, 4) and reverse primers specific either for the 5’-end of gusA (lanes 1, 3) or the 3’-end of gusA (lane 2, 4). Lane M, molecular size markers. (PPTX) [file ppat.1005502.s001.pptx]
