# Supplementary material for: A Functional Bacterium-to-Plant DNA Transfer Machinery of Rhizobium etli
Source: PLoS Pathog. 2016 Mar 11;12(3):e1005502. doi: 10.1371/journal.ppat.1005502 (PMC4788154; doi:10.1371/journal.ppat.1005502)
Supplement: S1 Table — Gray shading indicates proteins essential for Agrobacterium tumorigenicity [23]. OLN, ordered locus name represents the naming system for sequential assignment of an identifier to each predicted gene of a completely sequenced genome (http://www.uniprot.org/help/gene_name). Percentage of identity (Identity %) and percentage of query cover (Query Cover %) are indicated. Protein sequences were compared using the blastp program (PubMed) with the corresponding protein sequences of Agrobacterium tumefaciens strain C58 as reference. NS = no proteins with significant homology were identified. (DOCX) [file ppat.1005502.s002.docx]

| **Vir Protein** | **pTiC58**  **OLN** | **Species (Strain)** | **Identity %**  **(Query Cover %)** | ***Rhizobium***  **Protein ID** |
| --- | --- | --- | --- | --- |
| VirA | Atu6166 | *Rhizobium mesoamericanum* (STM3625) | 30 (34) | CCM78990.1 |
|  |  | *Rhizobium tropici* | NS |  |
|  |  | *Mesorhizobium loti* (R7A) | 45 (97) | CAD31451.1 |
| VirB1 | Atu6167 | *Rhizobium mesoamericanum* | 76 (97) | WP_007538755.1 |
|  |  | *Rhizobium tropici* | 29 (72) | WP_047635188.1 |
|  |  | *Mesorhizobium loti* (R7A) | 49 (97) | CAD31462.1 |
| VirB2 | Atu6168 | *Rhizobium mesoamericanum* (STM3625) | 85 (100) | CCM79764.1 |
|  |  | *Rhizobium tropici* | NS |  |
|  |  | *Mesorhizobium loti* (R7A) | 66 (100) | CAD31461.1 |
| VirB3 | Atu6169 | *Rhizobium mesoamericanum* (STM3625) | 44 (23) | CCM75065.1 |
|  |  | *Rhizobium tropici* | 38 (85) | WP_047639517.1 |
|  |  | *Mesorhizobium loti* (R7A) | 83 (99) | CAD31460.1 |
| VirB4 | Atu6170 | *Rhizobium mesoamericanum* (STM3625) | 24 (86) | CCM80092.1 |
|  |  | *Rhizobium tropici* | 27 (93) | WP_052213444.1 |
|  |  | *Mesorhizobium loti* (R7A) | 80 (100) | CAD31459.1 |
| VirB5 | Atu6171 | *Rhizobium mesoamericanum* (STM3625) | 83 (99) | CCM79761.1 |
|  |  | *Rhizobium tropici* | NS |  |
|  |  | *Mesorhizobium loti* (R7A) | 56 (98) | CAD31458.1 |
| VirB6 | Atu6172 | *Rhizobium mesoamericanum* | 76 (100) | WP_007538745.1 |
|  |  | *Rhizobium tropici* | 29 (73) | WP_047635212.1 |
|  |  | *Mesorhizobium loti* (R7A) | 70 (98) | CAD31457.1 |
| VirB7 | Atu6173 | *Rhizobium mesoamericanum* (STM3625*)* | 80 (89) | CCM79759.1 |
|  |  | *Rhizobium tropici* | NS |  |
|  |  | *Mesorhizobium loti* (R7A) | 57 (83) | CAD31456.1 |
| VirB8 | Atu6174 | *Rhizobium mesoamericanum* | 89 (100) | WP_007538740.1 |
|  |  | *Rhizobium tropici* | 25 (93) | WP_047635217.1 |
|  |  | *Mesorhizobium loti* (R7A) | 77 (100) | CAD31455.1 |
| VirB9 | Atu6175 | *Rhizobium mesoamericanum* | 86 (100) | WP_007538739.1 |
|  |  | *Rhizobium tropici* | 34 (98) | WP_047635220.1 |
|  |  | *Mesorhizobium loti* (R7A) | 69 (98) | CAD31454.1 |
| VirB10 | Atu6176 | *Rhizobium mesoamericanum* | 80 (100) | WP_028749564.1 |
|  |  | *Rhizobium tropici* | 36 (89) | WP_047617802.1 |
|  |  | *Mesorhizobium loti* (R7A) | 60 (99) | CAD31453.1 |
| VirB11 | Atu6177 | *Rhizobium mesoamericanum* (STM3625) | 91 (100) | CCM79755.1 |
|  |  | *Rhizobium tropici* | 42 (93) | WP_047617804.1 |
|  |  | *Mesorhizobium loti* (R7A) | 83 (99) | CAD31452.1 |
| VirC1 | Atu6180 | *Rhizobium mesoamericanum* (STM3625) | 96 (69) | CCM79579.1 |
|  |  | *Rhizobium tropici* | 70 (99) | WP_047618122.1 |
|  |  | *Mesorhizobium loti* | NS |  |
| VirC2 | Atu6179 | *Rhizobium mesoamericanum* (STM3625) | 88 (15) | CCM79580.1 |
|  |  | *Rhizobium tropici* | 37 (91) | WP_047618124.1 |
|  |  | *Mesorhizobium loti* | NS |  |
| VirD2 | Atu6182 | *Rhizobium mesoamericanum* | 59 (100) | WP_028749266.1 |
|  |  | *Rhizobium tropici* | NS |  |
|  |  | *Mesorhizobium loti* | NS |  |
| VirD3 | Atu6183 | *Rhizobium mesoamericanum* (STM3625) | 38 (6) | CCM79300.1 |
|  |  | *Rhizobium tropici* | NS |  |
|  |  | *Mesorhizobium loti* | NS |  |
| VirD4 | Atu6184 | *Rhizobium mesoamericanum* | 84 (98) | WP_007538682.1 |
|  |  | *Rhizobium tropici* | 62 (96) | WP_052213428.1 |
|  |  | *Mesorhizobium loti* | 84 (98) | WP_007538682.1 |
| VirD5 | Atu6185 | *Rhizobium mesoamericanum* (STM3625) | 26 (17) | CCM75345.1 |
|  |  | *Rhizobium tropici* | NS |  |
|  |  | *Mesorhizobium loti* | NS |  |
| VirE1 | Atu6189 | *Rhizobium mesoamericanum* | NS |  |
|  |  | *Rhizobium tropici* | NS |  |
|  |  | *Mesorhizobium loti* | NS |  |
| VirE2 | Atu6190 | *Rhizobium mesoamericanum* | 39 (5) | WP_028745412.1 |
|  |  | *Rhizobium tropici* | 23 (66) | WP_047618117.1 |
|  |  | *Mesorhizobium loti* | NS |  |
| VirE3 | Atu6191 | *Rhizobium mesoamericanum* | NS |  |
|  |  | *Rhizobium tropici* | NS |  |
|  |  | *Mesorhizobium loti* | NS |  |
| VirF | Atu6154 | *Rhizobium mesoamericanum* | 30 (90) | WP_007538761.1 |
|  |  | *Rhizobium tropici* | NS |  |
|  |  | *Mesorhizobium loti* | NS |  |
| VirG | Atu6178 | *Rhizobium mesoamericanum* (STM3625) | 88 (95) | CCM79754.1 |
|  |  | *Rhizobium tropici* | NS |  |
|  |  | *Mesorhizobium loti (R7A)* | 76 (95) | CAD31463.1 |
| VirH1 | Atu6150 | *Rhizobium mesoamericanum* (STM3625) | 26 (83) | CCM80358.1 |
|  |  | *Rhizobium tropici* | NS |  |
|  |  | *Mesorhizobium loti* | NS |  |
| VirH2 | Atu6151 | *Rhizobium mesoamericanum* (STM3625) | 23 (76) | CCM80357.1 |
|  |  | *Rhizobium tropici* | NS |  |
|  |  | *Mesorhizobium loti* | NS |  |
